# Perioperative management of a patient with unexpectedly detected early-stage ovarian mucinous carcinoma combined with progressive bulbar paralysis: a case report and literature review

**DOI:** 10.1186/s12905-024-03117-9

**Published:** 2024-05-04

**Authors:** Dingbei Zhang, Ruibo Xu, Tingting Huo, Ying Liu, Zengfang Hao, Yao Sun, Xiaoyu Xi, Xiaoli Du, Lili Wang, Jiexian Du

**Affiliations:** 1 Department of Gynecology, The Second Hospital of Hebei Medical University, Shijiazhuang, 050000 Hebei China; 2 Department of Gynecology, Handan first hospital, Handan, 056000 Hebei China; 3 Department of Anaesthesiology, First Teaching Hospital of Tianjin University of Traditional Chinese Medicine, Tianjin, 300072 China; 4 Department of Ultrasound, The Second Hospital of Hebei Medical University, Shijiazhuang, 050000 Hebei China; 5 Department of Pathology, The Second Hospital of Hebei Medical University, Shijiazhuang, 050000 Hebei China; 6 Department of Gynecology, Traditional Chinese Medicine Hospital of Shijiazhuang, Hebei, 050000 China

**Keywords:** Case report, Giant ovarian cysts (GOCs), Perioperative management, Progressive bulbar paralysis (PBP), Perioperative management, Multi-disciplinary treatment (MDT)

## Abstract

**Background:**

Giant ovarian cysts (GOCs)complicated with progressive bulbar paralysis (PBP) are very rare, and no such literature about these cases have been reported. Through the diagnosis and treatment of this case, the perioperative related treatment of such patients was analyzed in detail, and early-stage ovarian mucinous carcinoma was unexpectedly found during the treatment, which provided reference for clinical diagnosis and treatment of this kind of diseases.

**Case presentation:**

In this article, we reported a 38-year-old female patient. The patient was diagnosed with PBP 2 years ago. Examination revealed a large fluid-dominated cystic solid mass in the pelvis measuring approximately 28.6×14.2×8.0 cm. Carbohydrate antigen19-9(CA19-9) 29.20 IU/mL and no other significant abnormalities were observed. The patient eventually underwent transabdominal right adnexal resection under regional anesthesia, epidural block. Postoperative pathology showed mucinous carcinoma in some areas of the right ovary. The patient was staged as stage IA, and surveillance was chosen. With postoperative follow-up 1 month later, her CA19-9 decreased to 14.50 IU/ml.

**Conclusions:**

GOCs combined with PBP patients require a multi-disciplinary treatment. Preoperative evaluation of the patient's PBP progression, selection of the surgical approach in relation to the patient's fertility requirements, the nature of the ovarian cyst and systemic condition are required. Early mucinous ovarian cancer accidentally discovered after operation and needs individualized treatment according to the guidelines and the patient's situation. The patient's dysphagia and respiratory function should be closely monitored during the perioperative period. In addition, moral support from the family is also very important.

## Background

Ovarian cysts are the most common female pelvic masses, and when ovarian cysts are larger than 10 cm in diameter, they are called giant ovarian cysts (GOCs) [1]. GOCs can cause abdominal distention, pain, nausea and vomiting, and even intestinal obstruction and hydronephrosis.

Amyotrophic lateral sclerosis (ALS) is a rapidly progressive neurodegenerative disease of the human motor system, clinically characterized by upper and lower motor neuron dysfunction, with an incidence of 1.7/100,000 and a median survival of approximately 5 years after diagnosis [2]. Progressive bulbar palsy (PBP), or called bulbar phenotype ALS is mainly characterized by dysarthria and/or dysphagia. The median survival time of PBP was shorter than other subgroups. Since the patient's ovarian cancer was discovered unexpectedly, the article focuses on GOCs combined with PBP. PBP poses a great challenge to the perioperative management of patients with GOCs, so the purpose of this article is to discuss the perioperative management of patients with GOCs combined with PBP.

## Case presentation

**Fig. 1:**
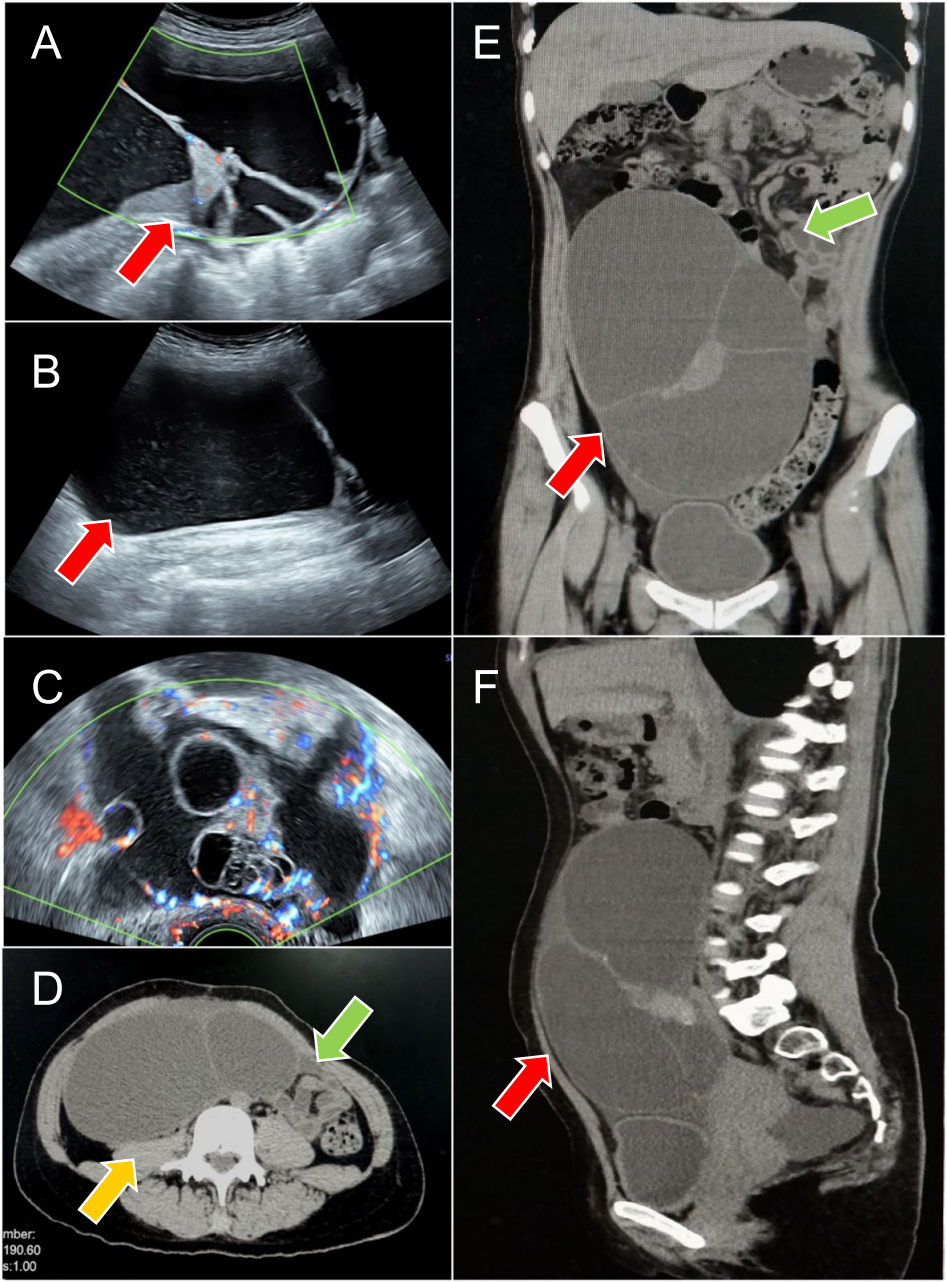
The red arrows represent pelvic and abdominal masses, this yellow arrow represents the compressed renal pelvis and the right ureter, and the green arrows represent the compressed intestine. Figures **A**-**C** are gynecological ultrasound images. Figures **A** and **B** show a giant fluid-dominated mass from the suprapubic area between the umbilicus and the glabella, reaching the anterior axillary line on both sides, with a size of about 28.6×14.2 cm. Figure **C**: color Doppler ultrasound showed no significant abnormal blood flow signal. Figure **D**-**F**: the size of the lesion was about 15.0×7.1×20.2 cm, with no significant enhancement of the cystic component and more uniform enhancement of the solid component after enhancement

A 38-year-old female is presented with a large pelvic mass. Gynecologic ultrasound showed a large fluid-dominated mass and measuring approximately 28.6×14.2×8.0 cm (Fig. 1A, B). Color Doppler ultrasound showed no particular abnormal blood flow signal (Fig. 1C). Computerized tomography (CT) showed slight dilatation of renal pelvis and right ureter, and compression of adjacent intestine. The lesion size was approximately 15.0×7.1×27.2 cm, with no significant enhancement of the cystic component and more uniform enhancement of the solid component (Fig. 1D-F). Carbohydrate antigen 19-9(CA19-9) 29.20 IU/mL, remaining examination were normal.

Two years ago, she developed pharyngeal discomfort, nausea while reading and brushing teeth, which gradually worsened. A year and a half ago, she had inflexible tongue movements, slurred speech, low pitch, choking and coughing. Patient did not take riluzole or other drugs. The neurological examination of this hospitalization said: poor dysarthria, tongue muscle atrophy, eye reflexes not elicited, muscle strength of both upper limbs grade 5-, muscle strength of both lower limbs grade 5. Pulmonary function tests were not performed because the patient had puffing and leaking air. The score of Amyotrophic lateral sclerosis functional rating scale revised (ALSFRS-R) was 44, indicating that most of the daily activities of the patient were not affected. The results of the SDS depression self-assessment scale showed a crude score of 32 and the standard score was 40, indicating that the patient was not depressed. The rest of the examination did not show any significant abnormalities.

With multi-disciplinary treatment (MDT) discussion before operation, the patient finally chose midline laparotomy and underwent right adnexectomy under regional anesthesia with an epidural gap block. The incision reaches above the umbilicus. Intraoperatively, atropine was used to suppress glandular secretion and inhibit saliva production, thereby reducing swallowing action. Lidocaine and ropivacaine can relieve pain, and the block plane reached T10, and the VAS score of tolerable pain during operation was 6. Intraoperative exploration revealed no ascites in the pelvic abdomen and an irregular cyst in the right ovary, approximately 30 cm in diameter, with an intact envelope and smooth surface. Right adnexal resection was performed after aspiration of some intracapsular fluid by puncture. The specimen had smooth walls, viscous intracapsular fluid, locally visible gelatinous tissue, but no cauliflower-like masses or papillae, and normal appearance of the right fallopian tube. During the operation, the patient and her family refused to have a complete staging operation.

**Fig. 2:**
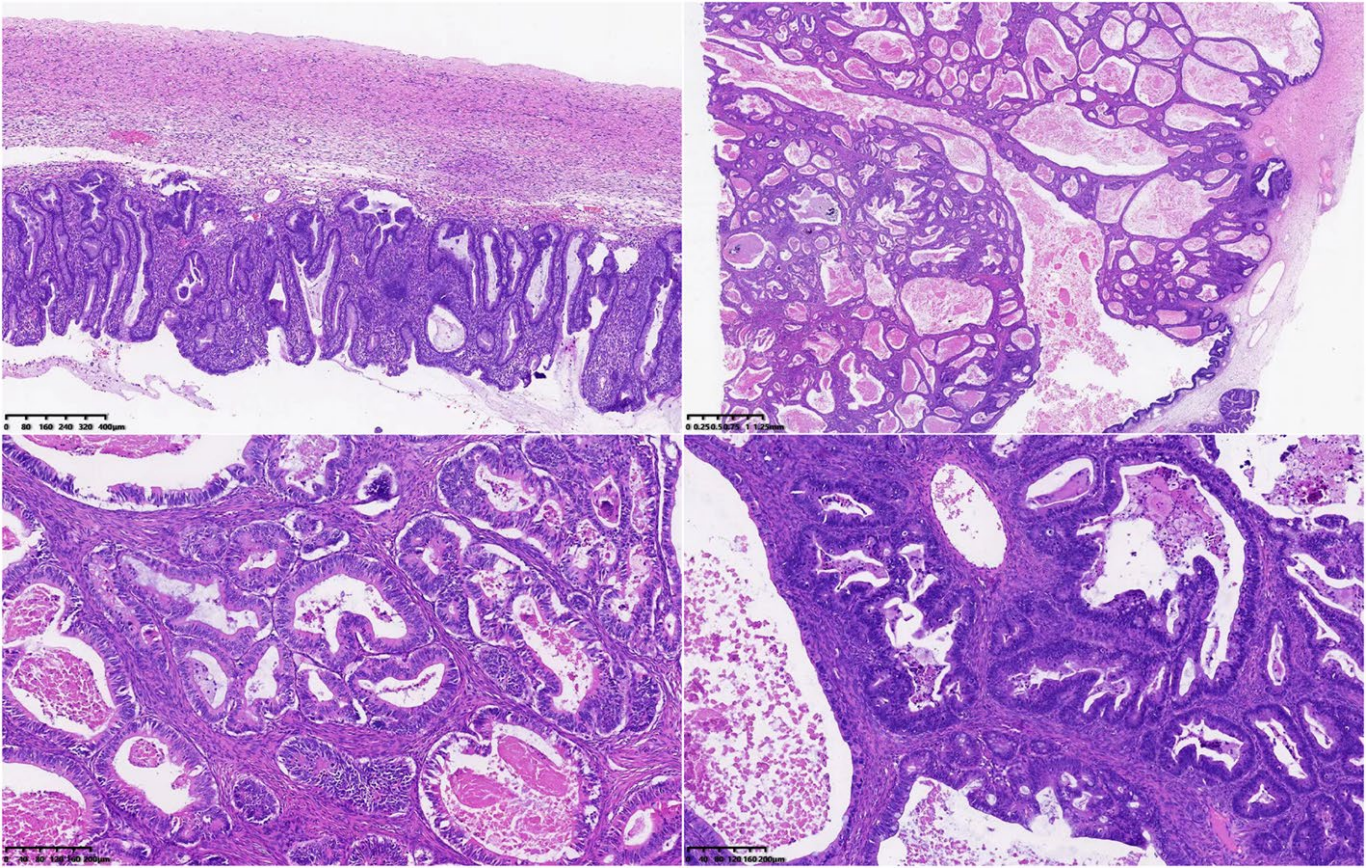
Postoperative pathology suggested (right ovary) junctional mucinous tumor with some areas showing mucinous carcinoma, the tumor size was about 15cm×10cm×5cm, no definite intravascular carcinoma embolus was seen: (right) fallopian tube did not show carcinoma

After operation, MDT found that the patient recovered well without nausea, vomiting, cough, sputum or dyspnea, and a tolerable pain VAS score of 8, along with good wound healing. And the symptoms of PBP did not worsen. The postoperative pathology suggested (right ovary) junctional mucinous tumor with some areas showing mucinous carcinoma and no clear intravascular carcinoma emboli: the (right) fallopian tube did not show carcinoma (Fig. 2). After consultation with the patient and family, it was decided to surveillance. One month after surgery, CA19-9 decreased to 14.50 IU/ml.

## Discussion

### Preoperative analysis

It is very important to judge the benign and malignant of GOCs before operation. According to the tumor markers of patients, Carbohydrate antigen 125 (CA125) and Human epididymal secretory protein 4 (HE4) are the most valuable tumor markers applied to ovarian cysts. Alpha-fetoprotein (AFP) and CA19-9 is also used to evaluate the nature of the tumor [3, 4]. The overall sensitivity of CA125 in differentiating benign and malignant tumors is 61% ~ 90%; The specificity was 71%~93%, the positive predictive value (PPV) was 35%~91%, and the negative predictive value (NPV) was 67%~90% [5]. HE4 is a potential biological marker for differentiating benign and malignant ovarian tumors. Combined examination of HE4 and CA125 can further improve the sensitivity and specificity of diagnosis of ovarian malignant tumor [6]. AFP is a specific marker of ovarian endodermal sinus tumor and can also be used in diagnosis. The patients reported in this report showed no abnormality in CA125, HE4 and AFP, while CA19-9 was slightly elevated. According to the values of CA125 and HE4, the calculated ROMA index is 7.341%, suggesting that ovarian cancer is of low risk and may be a benign tumor. Regarding the risk assessment methods of benign and malignant ovarian cysts, according to the rules of the International Ovarian Tumor Analysis Group (IOTA) [4], the rules consist of five characteristics indicating malignant lesions (M rule) and five characteristics indicating benign lesions (B rule). Among them, the malignant standard (M rule) is multilocular cystic solid lesion (maximum diameter ≥100mm、color score 2-3). Therefore, it has the characteristics of M and belongs to malignant tumor according to IOTA classification.

The neurologist needs a neurological examination, including muscle volume, muscle strength, swallowing function, speech function, and respiratory function. ALSFRSr includes four domains: medullary function, fine motor function, gross motor function and respiratory function, and is an important tool for functional assessment of ALS [7]. Forced vital capacity (FVC), maximum inspiratory pressure (MIP) can be a good measure of diaphragmatic strength. In patients with advanced or medullary involvement, due to difficulties in forming a tight seal around the mouth, collapse of the upper airway, and motor defects of the airway and upper airway muscles, sniffing nasal inspiratory pressure (SNIP) is a good measurement tool [8].

Anesthetic management must consider the effects on respiratory function. It has been shown that general anesthesia inhibits the excitability of spinal motor neurons while depressing the respiratory system, which may accelerate motor neuron disease after surgery [9]. Cases related to neurological sequelae after spinal anesthesia have been reported in the past, while the concentration of local anesthetics in the cerebrospinal fluid is much smaller during epidural anesthesia, and it seems prudent to use epidural anesthesia [10, 11].

The patient's ovarian tumor had compressed the renal pelvis and ureter, and malignancy could not be completely ruled out, so surgery was recommended. Since the patient had no fertility requirements and could not tolerate multiple surgeries, a right ovariectomy was planned. Because laparoscopic surgery requires the injection of carbon dioxide (CO2) into the abdominal cavity, resulting in intra-abdominal pressure (IAP) of 12 to 15 mmHg, increased IAP, displacement of the diaphragm cephalad, and decreased pulmonary compliance, this may lead to a ventilation-perfusion mismatch, resulting in hypoxemia, and this is further exacerbated in the trending supine position (hammerhead supine position). Abdominal distension may also lead to pulmonary complications such as hypercapnia and subcutaneous emphysema [12]. If we choose gasless laparoscopy, it may lead to the rupture of giant ovarian cyst during the operation, which will affect the prognosis. Attempted laparoscopic surgery is a great challenge for PBP patients. A combined intra-neurological and anesthetic evaluation of the patient for feasible surgery resulted in the choice of transabdominal right adnexal resection with epidural gap block.

### Precautions during operation

Hemodynamic instability is a worrisome complication of GOCs surgery. Large masses compress the vena cava, thereby reducing venous return, and further instability is caused by dilatation of the visceral vessels after mass resection [13, 14]. The masses should be removed slowly intraoperatively, and changes in blood volume should be closely monitored and managed promptly.

During epidural anesthesia, the plane of anesthesia should not be chosen too high (L12-S1~2 plane), otherwise it will affect the patient's respiratory function, and when the patient has pain intolerance during operation, analgesics are given promptly to relieve pain [10]. Concerning salivation, anticholinergic drugs and botulinum toxin can be used to reduce glandular secretion [15]. The early sensitivity of percutaneous blood oxygen saturation measurement is poor, and hypoxemia cannot be detected in time and should be judged by the results of arterial blood gas analysis.

Intraoperative frozen pathology should have been performed according to the guidelines, but the operative time in this patient should not have been too long, and the false-negative rate of diagnosis based on the absence of ascites during the operation and the absence of papillary projections on the inner wall of the ovarian cyst, as well as mucinous ovarian tumors and junctional tumors usually have a high rate of false-negative frozen section diagnosis [1, 3, 16]. After communication with the patient's family, the patient refused to send the frozen pathology during the operation.

### Postoperative management

Dysarthria and dysphagia occur in almost 80% of patients with medullary ALS [17]. Therefore, communication should be done by asking more questions that can be answered with yes or no. Dysphagia can lead to aspiration and malnutrition. High-calorie and high-protein oral nutritional supplements are good choices. Enteral nutrition is an appropriate intervention if the patient has lost more than 10% of his premorbid weight [17].

PBP causes weakness of the muscles of the mouth, face and tongue, which leads to impaired secretion clearance and impaired coughing, predisposes to respiratory infections, and increases morbidity and mortality. Chest CT can be performed to detect pulmonary infection and pulmonary atelectasis when the patient has symptoms such as fever, persistent cough and dyspnea. Anticholinergic drugs, botulinum toxin injections, improved overall hydration status, treatment with nebulizers, and increased environmental humidity all of which can help with secretion clearance.

ALS is a disease that strongly affects the psychiatric aspects of patients [8]. Caregivers and healthcare professionals should pay real-time attention to the psychological status of patients and provide positive emotional support. And that early postoperative activity is beneficial to patients, so it is acceptable to encourage patients to move appropriately after surgery [18, 19].

In conclusion, postoperative dysphagia, secretion clearance, and management of respiratory function are crucial, and postoperative emotional management should not be neglected; all postoperative conditions of the patient should be closely monitored and dealt with accordingly in a timely manner.

### Postoperative pathological results and follow-up treatment

Postoperative pathology reported mucinous carcinoma in some areas of the right ovary. Combining the patient's preoperative imaging, intraoperative situation and postoperative pathology findings, the patient was a stage IA ovarian mucinous carcinoma according to NCCN guidelines [20].

According to the NCCN guidelines: Patients have the option of reoperation: as patients have no fertility desire, a complete staging surgery is feasible. The procedure requires resection of the entire uterus, both adnexa, greater omentum, and appendix, with pelvic lymph node and para-aortic lymph node dissection [20, 21]. However, anesthesiologists assess that anesthesia for the procedure is difficult and extremely risky because the level of epidural anesthesia is too high and affects the patient's respiratory function, and that lymph node metastases from ovarian mucinous carcinoma are very rare. Combined with the patient's adjuvant findings, the tumor stage is likely to remain unchanged after surgery [22]. The average survival for ALS is 3-5 years [17]. Surgery is not the best option for the patient, and surveillanc is chosen by the patient and family after deliberation.

## Conclusion

GOCs combined with PBP is very rare. The perioperative management of patients with GOCs combined with PBP requires the combined efforts of neurologists, gynecologists, and anesthesiologists. Preoperatively, the patient's PBP progression is assessed and the anesthetic and surgical approach is felt in relation to the patient's fertility requirements, the nature of the ovarian cyst and general condition. As patients with PBP mainly present with dysarthria and dysphagia, epidural anesthesia was chosen as the mode of anesthesia. Postoperative patients should pay close attention to possible complications of ALS, especially dysphagia and active respiratory function, and timely carry out corresponding treatment. Also, positive emotional supporting is very important. Since ovarian cysts have the possibility of malignancy and simple resection of ovarian cysts or unilateral adnexal resection is feasible, prompt surgical treatment is recommended for patients with ovarian cysts combined with PBP to prevent malignancy.

## Data Availability

Submission of a manuscript to a BMC journal implies that materials described in the manuscript, including all relevant raw data, will be freely available to any scientist wishing to use them for non-commercial purposes, without breaching participant confidentiality. All the data of this study are presented in the article, and those who need it can use it directly, as long as the source is indicated. If you have any questions, please contact the corresponding author, e-mail: dujiexian2009@163.com

## Abbreviations

ALS: amyotrophic lateral sclerosis
GOCs: giant ovarian cysts
PBP: progressive bulbar paralysis
CT: computerized tomography
CA125: carbohydrate antigen 125
HE4: Human epididymal secretory protein 4
AFP: Alpha-fetoprotein
CA19-9: carbohydrate antigen 19-9
FVC: Forced vital capacity
MIP: maximum inspiratory pressure
SNIP: sniffing nasal inspiratory pressure
CO2: carbon dioxide
IAP: intra-abdominal pressure

## References

[1] Jiang, L., Zhao, X., Han, Y., Liu, K., Meng, X. J. F. i. o., Giant Ovarian Cysts Treated by Single-Port Laparoscopic Surgery: A Case Series. 2021;11:796330. 10.3389/fonc.2021.796330 PMC8695676 34956907

[2] van den Bos MAJ, Geevasinga N, Higashihara M, Menon P, Vucic S (2019). Pathophysiology and Diagnosis of ALS: Insights from Advances in Neurophysiological Techniques. Int J Mol Sci.

[3] Reiser E, Pils D, Grimm C, Hoffmann I, Polterauer S, Kranawetter M, Aust S (2022). Defining Models to Classify between Benign and Malignant Adnexal Masses Using Routine Laboratory Parameters. Cancers..

[4] Timmerman, D.; Planchamp, F.; Bourne, T.; Landolfo, C.; du Bois, A.; Chiva, L.; Cibula, D.; Concin, N.; Fischerova, D.; Froyman, W.; Gallardo Madueño, G.; Lemley, B.; Loft, A.; Mereu, L.; Morice, P.; Querleu, D.; Testa, A.; Vergote, I.; Vandecaveye, V.; Scambia, G.; Fotopoulou, C. J. I. j. o. g. c. o. j. o. t. I. G. C. S. ESGO/ISUOG/IOTA/ESGE Consensus Statement on pre-operative diagnosis of ovarian tumors. 2021;31(7):961-982. 10.1136/ijgc-2021-002565 PMC8273689 34112736

[5] Dolgun ZN, Kabaca C, Karateke A, Iyibozkurt C, Inan C, Altintas AS, Karadag C (2017). The Use of Human Epididymis 4 and Cancer Antigen 125 Tumor Markers in the Benign or Malignant Differential Diagnosis of Pelvic or Adnexal Masses. Balkan Med J.

[6] Granato T, Porpora MG, Longo F, Angeloni A, Manganaro L, Anastasi E (2015). HE4 in the differential diagnosis of ovarian masses. Clin Chim Acta.

[7] Grad LI, Rouleau GA, Ravits J, Cashman NR (2017). Clinical Spectrum of Amyotrophic Lateral Sclerosis (ALS). Cold Spring Harb Perspect Med.

[8] Rosa Silva JP, Santiago Junior JB, Dos Santos EL, de Carvalho FO, de Franca Costa IMP, Mendonca DMF (2020). Quality of life and functional independence in amyotrophic lateral sclerosis: A systematic review. Neurosci Biobehav Rev.

[9] Pinto S, Swash M, de Carvalho M (2014). Does surgery accelerate progression of amyotrophic lateral sclerosis?. J Neurol Neurosurg Psychiatry.

[10] Gu J, Lin X (2017). Anesthesia and postoperative analgesia for a patient with amyotrophic lateral sclerosis. Minerva Anestesiol.

[11] Kock-Cordeiro D., Brusse, E., van den Biggelaar R., Eggink, A., van der Marel C. J. I. j. o. o. a., Combined spinal-epidural anesthesia with non-invasive ventilation during cesarean delivery of a woman with a recent diagnosis of amyotrophic lateral sclerosis. 2018;36:108-110. 10.1016/j.ijoa.2018.06.001 30017643

[12] Atkinson TM, Giraud GD, Togioka BM, Jones DB, Cigarroa JE (2017). Cardiovascular and Ventilatory Consequences of Laparoscopic Surgery. Circulation.

[13] Atkinson T, Giraud G, Togioka B, Jones D, Cigarroa JJC (2017). Cardiovascular and Ventilatory Consequences of Laparoscopic Surgery. Circulation.

[14] Van Damme L., De Waele J. J. C. c. Effect of decompressive laparotomy on organ function in patients with abdominal compartment syndrome: a systematic review and meta-analysis. Crit Care. 2018;22(1):179. 10.1186/s13054-018-2103-0 PMC6060511 30045753

[15] James E, Ellis C, Brassington R, Sathasivam S, Young CA (2022). Treatment for sialorrhea (excessive saliva) in people with motor neuron disease/amyotrophic lateral sclerosis. Cochrane Database Syst Rev.

[16] Lycke M, Kristjansdottir B, Sundfeldt K (2018). A multicenter clinical trial validating the performance of HE4, CA125, risk of ovarian malignancy algorithm and risk of malignancy index. Gynecol Oncol.

[17] Burgos R, Breton I, Cereda E, Desport JC, Dziewas R, Genton L, Gomes F, Jesus P, Leischker A, Muscaritoli M, Poulia KA, Preiser JC, Van der Marck M, Wirth R, Singer P, Bischoff SC (2018). ESPEN guideline clinical nutrition in neurology. Clin Nutr.

[18] Seeber AA, Pols AJ, Hijdra A, Grupstra HF, Willems DL, de Visser M (2019). Advance care planning in progressive neurological diseases: lessons from ALS. BMC Palliat Care.

[19] Brent, J. R., Franz C. K., Coleman J. M., 3rd; Ajroud-Driss, S., ALS: Management Problems. Neurol Clin 2020;38(3)565-575. 10.1016/j.ncl.2020.03.013 PMC7963888 32703469

[20] Armstrong D. K., Alvarez R. D., Bakkum-Gamez J. N., Barroilhet L., Behbakht K., Berchuck A., Chen L. M., Cristea M., DeRosa M., Eisenhauer E. L., Gershenson D. M., Gray H. J., Grisham R., Hakam A., Jain A., Karam A., Konecny G. E., Leath C. A., Liu J., Mahdi H., Martin L., Matei D., McHale M., McLean K., Miller D. S, O'Malley D. M. Percac-Lima, S., Ratner E, Remmenga S. W., Vargas, R., Werner T. L., Zsiros, E., Burns, J. L., Engh, A. M., Ovarian Cancer, Version 2.2020, NCCN Clinical Practice Guidelines in Oncology. J Natl Compr Canc Netw. 2021;19 (2):191-226. 10.6004/jnccn.2021.0007 33545690

[21] Lokich E, Palisoul M, Romano N, Craig Miller M, Robison K, Stuckey A, DiSilvestro P, Mathews C, Granai CO, Lambert-Messerlian G, Moore RG (2015). Assessing the risk of ovarian malignancy algorithm for the conservative management of women with a pelvic mass. Gynecol Oncol.

[22] Heyward QD, Nasioudis D, Cory L, Haggerty AF, Ko EM, Latif N (2021). Lymphadenectomy for early-stage mucinous ovarian carcinoma. Int J Gynecol Cancer.

